# Effects of Supplementation with Omega-3 and Omega-6 Polyunsaturated Fatty Acids and Antioxidant Vitamins, Combined with High-Intensity Functional Training, on Exercise Performance and Body Composition: A Randomized, Double-Blind, Placebo-Controlled Trial

**DOI:** 10.3390/nu16172914

**Published:** 2024-09-01

**Authors:** Georgios Posnakidis, Christoforos D. Giannaki, Vassilis Mougios, Marios Pantzaris, Ioannis Patrikios, Philip C. Calder, Dina K. Sari, Gregory C. Bogdanis, George Aphamis

**Affiliations:** 1 Department of Life and Health Sciences, University of Nicosia, Nicosia 2417, Cyprus; posnakidisgeorgios@gmail.com (G.P.); aphamis.g@unic.ac.cy (G.A.); 2 Research Centre for Exercise and Nutrition (RECEN), Nicosia 2417, Cyprus; 3 Department of Nutrition, Faculty of Medicine, Universitas Sumatera Utara, Medan 20155, Indonesia; dina@usu.ac.id; 4 Laboratory of Evaluation of Human Biological Performance, School of Physical Education and Sport Science at Thessaloniki, Aristotle University of Thessaloniki, 54124 Thessaloniki, Greece; mougios@auth.gr; 5 The Cyprus Institute of Neurology and Genetics, Nicosia 2371, Cyprus; pantzari@cing.ac.cy; 6 School of Medicine, European University Cyprus, Nicosia 2404, Cyprus; i.patrikios@euc.ac.cy; 7 Faculty of Medicine, University of Southampton, Southampton SO16 6YD, UK; p.c.calder@soton.ac.uk; 8 NIHR Southampton Biomedical Research Centre, University Hospital Southampton NHS Foundation Trust and University of Southampton, Southampton SO16 6YD, UK; 9 School of Physical Education and Sport Science, National and Kapodistrian University of Athens, 17237 Athens, Greece; gbogdanis@phed.uoa.gr

**Keywords:** muscle mass, hypertrophy, body fat, strength, physical performance, Neuroaspis PLP10, PUFA

## Abstract

The aim of this study was to investigate the effects of a supplement rich in ω-3 and ω-6 polyunsaturated fatty acids (PUFAs) and antioxidant vitamins on physical performance and body composition following a period of high-intensity functional training (HIFT). Nineteen healthy young adults (nine males, ten females) underwent an 8-week HIFT program (3 days·week^−1^) where they were randomized 1:1 into either the supplement group (SG)—*n* = 10, receiving a 20 mL daily dose of a dietary cocktail formula (Neuroaspis™ PLP10) containing a mixture of ω-3 and ω-6 PUFAs (12,150 mg), vitamin A (0.6 mg), vitamin E (22 mg), and γ-tocopherol (760 mg)—or the placebo group (PG)—*n* = 9, receiving a 20 mL daily dose of virgin olive oil. Body composition, cardiorespiratory fitness, muscle strength, and muscle endurance were assessed before and after the training period. Body mass did not change, but muscle mass increased by 1.7 ± 1.9% or 0.40 ± 0.53 kg in the SG (*p* = 0.021) and decreased by 1.2 ± 1.6% or 0.28 ± 0.43 kg (*p* = 0.097) in the PG, compared with baseline. VO_2_max, vertical jump, squat 1RM, bench press 1RM, and muscle endurance increased similarly in both groups. The effects of HIFT on physical performance parameters, muscle damage, and inflammation indices were not affected by the supplementation. In conclusion, HIFT combined with high doses of ω-3 and ω-6 PUFAs and antioxidant vitamins resulted in a small but significant increase in muscle mass and fat reduction compared with HIFT alone.

## 1. Introduction

High-intensity functional training (HIFT) programs consist of a circuit of functional, multijoint movements [1] that may include bodyweight or resistance exercises, aerobic components, and other high-intensity activities performed over a short period of time, which is usually around 30 min [2,3]. This format of high-intensity exercise is very popular among recreational individuals and can be conducted either in groups or on an individual basis. [4]. HIFT has been shown to improve cardiorespiratory fitness, reduce body fat, and improve body composition and muscle power [5]. Muscle endurance has also been shown to improve with HIFT [6], and HIFT may induce various degrees of muscle mass and strength gains, depending on exercise format and resistive load [7,8]. Furthermore, high-intensity exercise has been shown to be beneficial for health-related parameters, as it reduces total serum cholesterol and low-density lipoprotein cholesterol and improves various quality-of-life components [9,10].

Whilst the benefits of high-intensity exercise and HIFT on body composition and cardiovascular function and capacity are well documented [3,6,8], a potential ergogenic effect of ω-3 or ω-6 polyunsaturated fatty acids (PUFAs) in HIFT is still unexplored. Ω-3 PUFAs can integrate into mitochondrial membranes and improve mitochondrial function [11]. Ω-3 PUFA supplementation can improve aerobic endurance, especially at submaximal intensities, due to a reduced oxygen cost [12], and may even lead to increases in VO_2_max [13], although this finding is not consistent [14]. Studies investigating the effects of ω-3 PUFAs on muscle strength have shown an increase in muscle protein synthesis [15] and a potential increase in strength [16] in older adults. Since HIFT combines aerobic and strength exercises, it is of great interest to investigate the potential effects of ω-3 PUFA supplementation during HIFT. Notably, the available data in the literature regarding the effectiveness of omega-6 on parameters of exercise performance and physical fitness are clearly fewer compared to omega-3. With regard to ω-6, there is information in the literature that a high ω-6 intake may have negative effects under certain circumstances. An excess of ω-6 in relation to ω-3 poses a risk for long-term inflammatory disorders [17]. Studies have shown that ill effects can manifest at a high ω-6:ω-3 supplementation ratio of 20:1 [18], whereas an ω-6:ω-3 PUFA supplementation ratio of 0.5–1.0 [19] appears to offer a protective effect. This information has been taken into consideration when selecting the nutritional supplement for the present study, at a favorable ω-6:ω-3 ratio of 1:0.52.

In recent years, there has been significant interest in the impact of PUFAs on body composition. Findings from recent systematic reviews and meta-analyses have indicated that the administration of omega-3 polyunsaturated fatty acids may enhance muscle protein synthesis [20] and overall body muscle mass and strength [21]. However, the effect of PUFA in combination with high-intensity exercise on body composition remains uncertain.

Another factor to consider during high-intensity exercise is the degree of stress posed on the immune system. For instance, exercising at a high percentage of VO_2_max may elicit greater inflammatory responses and muscle damage compared to a moderate intensity [22]. Creatine kinase (CK), as an index of muscle fiber damage, and C-reactive protein (CRP), as an index of systemic inflammation, may increase after both moderate- and high-intensity exercise, with higher values observed following the latter [23]. A certain degree of exercise-induced inflammation is now considered normal and expected, as this process stimulates muscle repair and adaptations during regular training [24]. However, excessive inflammation and muscle damage may have negative effects, and, thus, controlling the degree of inflammation is essential [23]. There are indications that nutritional manipulations or certain supplements might aid in this regard [25]. For instance, ω-3 PUFA supplementation may support recovery processes after exercise and have a beneficial effect on cardiovascular and muscular performance and health [26].

An attenuated oxidative stress response is another factor to consider when performing HIFT. Oxidative stress is considerably increased at intensities over 70% VO_2_max [27] and more so during and after high-intensity interval exercise [28]. Thus, the oxidative stress response to HIFT must be taken into account when planning HIFT sessions for non-athlete individuals [27]. Supplementation with antioxidant vitamins, such as vitamins C and E, has been shown to impair gains in lean mass following resistance training [29] and hinder adaptations to endurance training [30], although a recent systematic review and meta-analysis concluded that these vitamins do not appear to negatively affect VO_2_max [31]. Therefore, the aim of the present study was to investigate the effects of a nutritional supplement containing ω-3 and ω-6 PUFAs and antioxidant vitamins, consumed during an 8-week HIFT period, on strength and aerobic performance, as well as on body composition.

## 2. Materials and Methods

### 2.1. Participants and Ethics

Initially, twenty healthy young adults volunteered for the present study, but one dropped out before randomization for personal reasons. Nineteen participants (ten females) completed the study. Due to the demanding nature of HIFT, all participants were familiar with this form of exercise, had at least 6 months of experience in similar exercise programs, and were examined by a medical doctor before joining the study. Following baseline measurements, participants were randomly assigned (1:1) according to their sex, body mass and composition, and aerobic capacity to either the experimental supplement group (SG, *n* = 10, 29 ± 6 years, 5 females) or the placebo group (PG, *n* = 9, 30 ± 3 years, 5 females) using the lottery method, performed by an independent individual not related to the investigating team. An a priori power analysis for mixed-model ANOVA interaction (group × time) was conducted to determine the minimum sample size required based on a power of 0.80, alpha of 0.05, and correlation coefficient of 0.6 between repeated measures (G-Power software, v. 3.1.9.2, Universität Kiel, Kiel, Germany). In a recent systematic review, the effect size regarding whole-body protein synthesis after Omega-3 supplementation ranged between medium and large [20]. Therefore, an effect size within this range (η^2^ = 0.1) was used in the a priori power analysis, which yielded 9 participants per group. The study was approved by the Cyprus National Bioethics Committee (CNBC/2016/56; 21 March 2017), and the trial was registered with the ACTRN Clinical Trials Registry (ACTRN12624001044516). All participants signed an informed consent form following a written and verbal explanation of the nature, aim, and methods of the study.

### 2.2. Study Design

**Figure 1:**
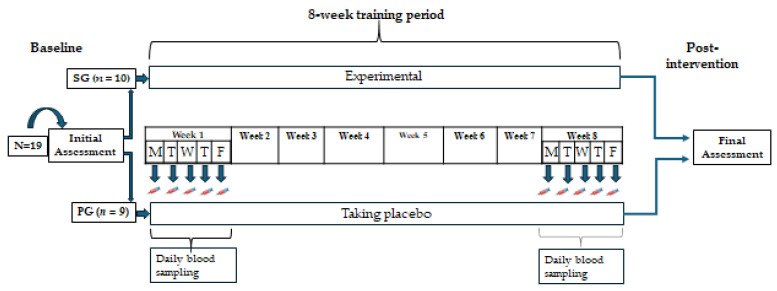
Experimental protocol of the study. SG, supplement group; PG, placebo group.

The study design is depicted in Figure 1. Participants of both groups trained together three times per week (Monday, Wednesday, and Friday) and followed the same HIFT protocol for 8 weeks. In the week preceding (baseline) and the week following the training period (post-intervention), all participants underwent physical performance and body composition assessment, investigating anthropometry, body composition, maximal oxygen uptake (VO_2_max), upper body strength, flexibility, and muscular endurance. All tests took place in the morning, except for the bench press 1RM and muscular endurance tests, which took place in the afternoon. During each workday of the first and last weeks of the training period, fasting blood samples were drawn to determine CK and CRP concentrations. The fatty acid composition of Red Blood Cell membranes (RBC) was assessed for compliance with the supplemented formulation.

All participants were asked to refrain from strenuous exercise on the day before the tests. Detailed food and fluid intake records were kept for 24 h before their first visit to the lab, and the diet was replicated before the post-training tests. Post-training tests took place at least 72 h following the last training session to allow for adequate recovery.

During the study, the SG consumed a specific supplement formula (20 mL per day for 8 weeks), rich in eicosapentaenoic acid (EPA) + docosahexaenoic acid (DHA) (6.9 g ω-3 PUFAs) and linoleic acid (LA) + γ-linolenic acid (GLA) (5.85 g ω-6 PUFAs) with vitamin E and gamma-tocopherol as antioxidant vitamins (Neuroaspis PLP10, Palupa Medical, Nicosia, Cyprus), and the PG consumed extra virgin olive (20 mL per day for 8 weeks). Both regiments had similar taste, color, odor, and texture. The supplement’s composition is described in further detail below.

### 2.3. Anthropometry and Body Composition

Height was measured with a stadiometer to the nearest 0.5 cm to calculate body mass index. All other anthropometric and body composition variables, that is, body mass (kg), body fat (%), and lean body mass (kg), were measured with a medical scale and body composition analyzer, based on multifrequency bioelectrical impedance analysis (Seca mBCA 515; Seca, Chino, CA, USA). All these measurements were taken in the early morning hours (7 a.m.) in a fasted state. The participants wore only light shorts and a T-shirt.

### 2.4. Cardiorespiratory Fitness

VO_2_max and maximum heart rate (HRmax) were measured using an incremental running protocol on a level treadmill (h/p/cosmos pulsar 3p; HP Cosmos, Nussdorf-Traunstein, Germany). Starting at 8 km·h^−1^, the running speed was increased every minute by 1 km·h^−1^ until exhaustion. Oxygen uptake was measured by a breath-by-breath analyzer (Quark CPET; Cosmed, Rome, Italy). Heart rate (HR) was continuously monitored using a telemetric heart rate monitor (H10 Heart Rate sensor; Polar, Kempele, Finland). All subjects achieved a true VO_2_max, as they met the criteria set by ACSM [32].

### 2.5. Vertical Jump Performance

The participants performed squat jumps (SJs), with their hands held firmly at their waist, and countermovement jumps (CMJs), using arm swings. Jump height was measured using Opto Jump Next (Microgate, Bolzano, Italy). Three efforts were allowed for each jump with a 1 min rest in between, and the best effort was recorded.

### 2.6. Upper Body Strength

Bench press was used to evaluate upper body strength. Since all participants had some experience, an estimate of their 1-repetition maximum (1RM) was used to set the weight for the initial sets of the bench press test. For warm-up, the participants performed 10 repetitions with low weights, and this was followed by a 2 min rest. Then, the weight was increased to an intensity of 50% of the estimated 1RM and the participants performed another 10 repetitions. With a 4 min rest between each set, the weight increased to 75% of the estimated 1RM for 5 repetitions, and then was set at 85% of the estimated 1RM for 3 repetitions. Thereafter, the weight increased gradually by 5 or 2.5 kg, with the participants performing 1 repetition at each weight until the true 1RM was achieved [33].

### 2.7. Muscle Endurance

Following the determination of their 1RM, the participants were asked to perform as many repetitions as possible at 65% 1RM with the correct technique. The test was terminated when the participants could not lift the bar or when their technique failed and they required assistance to lift the bar. The number of repetitions was used as a measure of upper body muscle endurance.

To determine the endurance of their abdominal muscles, the participants were asked to perform as many sit-ups as possible in one minute. Each participant lay on the ground, with their knees at a 90° angle, feet held firmly on the ground by an examiner, and arms crossed at the chest.

### 2.8. Biochemical Parameters

During each workday of the first and last weeks of the training period, 7.5 mL of fasting venous blood samples was drawn. Of those, 5 mL was dispensed into a plain tube for CK and CRP analyses. The remaining 2.5 mL was dispensed in an EDTA tube for RBC fatty acid analyses confirming compliance to the formula consumption. CRP and CK were measured using a COBAS INTEGRA 400 plus an automated analyzer (Roche Diagnostics Ltd., Rotkreuz, Switzerland). The fatty acid analysis was performed using gas chromatography (GC) following a standard method [34].

### 2.9. HIFT Protocol

Before the intervention, all participants underwent a familiarization session with the proposed exercise protocol. Emphasis was given to proper technique, safety, and the correct handling of the exercise equipment. During the exercise sessions, there was encouragement from the exercise scientist who supervised the program for the participants to give effort according to their abilities.

A standardized 10 min warm-up of the participants was carried out before the main HIFT protocol. The main HIFT session included four rounds of a 9-exercise circuit. The exercise-to-rest ratio was 30:15 s. A two-minute break was allowed after the completion of round 2 (mid-session point). The exercise protocol included the following exercises: (1) upright and sumo squats, (2) abdominal crunches whilst holding a medicine ball, (3) the clean and press, (4) box jumps, (5) TRX chest presses, (6) wallball throws, (7) burpees, (8) sledgehammers, and (9) repeated 10 m sprints. The resistance used for the weight-bearing exercises was 60% of 1RM. The medicine ball weights were 3 and 4 kg.

HR was continuously monitored and recorded to determine exercise intensity using the Polar Team H7 sensors and software (Polar Team, version 1.2).

All participants were asked to abstain from other strenuous activities during the duration of the study. To the best of our knowledge, the participants adhered to this instruction.

### 2.10. Supplementation

The daily oral dose of Neuroaspis plp10 dietary supplement was 20 mL and contained a cocktail mixture of EPA (about 1650 mg)/DHA (about 4650 mg)/GLA (about 2000 mg)/LA (about 3850 mg)/total other omega-3 (about 600 mg)/total monounsaturated fatty acids (about 1700 mg) + total saturated fatty acids (18:0 about 160 mg, 16:0 about 650 mg)/vitamin A (about 0.6 mg)/vitamin E (about 22 mg) and pure γ-tocopherol (760 mg). Subjects were requested to consume the supplement or a placebo half an hour before dinner.

The placebo was composed of pure virgin olive oil and was identical in color, smell, shape, size, and packaging to the Neuroaspis plp10, and both were bottled in dark bottles under a nitrogen bed. Both interventions contained a food-grade citrus aroma (~3.5 mL) for palatability and taste reasons. The bottles were labeled with medication code numbers that were unidentifiable for participants as well as investigators. Both Neuroaspis plp10 and the placebo were manufactured in Greece [34]. The participants of the placebo group consumed the same 20 mL dosage. Both the supplement and the placebo were in identical bottles and were administrated per os once daily, 30 min before dinner. Both the participants and the investigators were blind as to the content of the bottles.

### 2.11. Statistical Analysis

All statistical analyses were performed using SPSS for Windows, v.24.0 (IBM, Armonk, NY, USA). Descriptive data are presented as the means ± SD. A two-way, time × group, analysis of variance (ANOVA) with repeated measures on time was used to compare anthropometric, body composition, and performance variables before and after the exercise and nutritional interventions between groups. Additionally, a three-way, week × day × group ANOVA with repeated measures on week and day was used to analyze CK and CRP values. When a significant interaction was found, Tukey’s post hoc test was used to locate significant differences. Student’s *t*-test was used for the fatty acid analyses. The level of statistical significance was set at α = 0.05.

## 3. Results

### 3.1. Anthropometry and Body Composition

Anthropometric and body composition data are presented in Table 1. There was no time effect or group × time interaction for either body mass or BMI (Table 1). However, there were changes in body composition. Statistical analysis revealed a time × group interaction (*p* = 0.007, η^2^ = 0.351) for muscle mass. Post hoc testing showed that at baseline, there was no difference in muscle mass between groups (*p* = 0.516). After the training period, muscle mass increased by 1.7 ± 1.9% or 0.40 ± 0.53 kg in the SG (*p* = 0.021) and decreased by 1.2 ± 1.6% or 0.28 ± 0.43 kg (*p* = 0.097) in the PG, compared with baseline. The ANOVA also showed a time × group interaction for body fat (*p* = 0.03, η^2^ = 0.249). The post hoc test showed that there was a fat loss only in the SG by 2.0 ± 1.5% or 1.3 ± 1.1 kg (*p* = 0.003), while there was no change in body fat in the PG (*p* = 0.920).

### 3.2. Cardiorespiratory Parameters (Table 2)

The HIFT protocol resulted in significant improvements in VO_2_max (time effect *p* < 0.001, η^2^ = 0.839) for both groups (SG = 7.1%, PG = 5.5%), but there was no group (*p* = 0.241, η^2^ = 0.167) or time × group (*p* = 0.342, η^2^ = 0.113) effect. There was also a positive time effect on MAS (*p* < 0.001), as both groups improved (SG = 4%, and PG = 6%) without a group (*p* = 0.078) or time × group (*p* = 0.579) effect. There was no effect on HRmax during the intervention period (time effect *p* = 0.716, η^2^ = 0.017; group effect *p* = 0.475, η^2^ = 0.065; time × group *p* = 0.464, η^2^ = 0.069).

**Table 2:** Physical Performance Parameters.

Variables	SG (*n* = 10)	Change (%)	PG (*n* = 9)	Change (%)	Main Effects and Interactions (*p*-Value)		
					Time	Group	Time × Group
VO_2_ max (mL·kg^−1^·min^−1^)							
Before	46.95 ± 5.24		42.84 ± 9.48 *		0.073		
After	50.29 ± 4.83	7.1%	45.18 ± 9.30 *	5.5%	<0.001	0.241	0.342
HRmax (beats·min^−1^)							
Before	187 ± 10		188 ± 7		0.270		
After	186 ± 6	−0.5%	189 ± 7	0.5%	0.716	0.475	0.464
SJ (cm)							
Before	28.3 ± 5.8		26.5 ± 7.7 *		0.319		
After	32.7 ± 8.1	15.5%	29.5 ± 7.5 *	11.3%	<0.001	0.562	0.506
CMJ (cm)							
Before	33.8 ± 7.7		31.5 ± 9.5 *		0.438		
After	38.1 ± 9.0	12.7%	34.2 ± 9.75 *	8.6%	<0.001	0.577	0.204
Bench Press 1 RM (kg)							
Before	59 ± 21		43 ± 23 *		0.977		
After	62 ± 23	5.1%	48 ± 22 *	11.6%	<0.001	0.264	0.065
Bench press endurance, 65% 1 RM (reps)							
Before	21 ± 5		19 ± 5 *		0.697		
After	23 ± 2	9.5%	22 ± 4 *	15.8%	0.024	0.748	0.911
Sit-ups in 1 min (reps)							
Before	43 ± 5.2		37 ± 8.8 *		0.094		
After	49 ± 7.4	22.5%	44 ± 8.1 *	18.9%	<0.001	0.137	0.853

SG, experimental supplement group; PG, placebo group; SJ, squat jump; CMJ, countermovement jump; RM, repetition maximum. * Denotes difference from baseline within the group.

### 3.3. Strength and Muscle Endurance Parameters (Table 2)

Explosive power improved over time in both groups. For squat jumps, there was a time effect (*p* < 0.001, η^1^ = 0.860) as both groups increased their jump (SG 15.5%, PG 11.3%), but there was no group (*p* = 0.562, η^1^ = 0.044) or time × group effect (*p* = 0.506, η^1^ = 0.057). Similar changes were seen for CMJs, as both groups improved over time (SG 12.7%, PG 8.6%; *p* < 0.001, η^1^ = 0.849) without any group (*p* = 0.577, η^1^ =0.041) or time × group (*p* = 0.204, η^1^ = 0.193) effect.

In a similar fashion, with both groups having similar values at baseline, all other strength-related parameters improved over time. For bench press 1RM, there was a time effect (*p* = 0.002, η^1^ = 0.721) as both groups improved (SG 5.1%, PG11.6%) without any statistical group (*p* = 0.242, η^1^ = 0.166) or time × group effect (*p* = 0.065, η^1^ = 0.364). For bench press endurance at 65% 1RM, both groups again improved over time (SG 9.5%, PG 15.8%; time effect *p* = 0.024, η^1^ = 0.493) without a group (*p* = 0.748, η^1^ = 0.014) or time × group effect (*p* = 0.911, η^1^ =0.002). For sit-ups, in 1 min, there was a time effect and an improvement in both groups (SG 22.5%, PG 18.9%, *p* < 0.001, η^1^ = 0.813) without a group (*p* = 0.134, η^1^ = 0.254) or time × group effect (*p* = 0.853, η^1^ = 0.005).

### 3.4. Biochemical Parameters

The data regarding CK and CRP were analyzed for group effects (supplement vs. placebo), comparisons between the first and the eighth weeks of the exercise period, and weekday effects (Table 3). CK was similar between groups (*p* = 0.444) for both the first and eighth weeks of the training period (SG = 209 ± 33 vs. PG = 171 ± 35 U·L^−1^). Comparisons between the first and last weeks of the exercise program showed that total CK activity decreased similarly during the 8th week of the HIFT intervention in both groups (week effect *p* = 0.009, η^2^ = 0.339), as there was no group × week interaction (*p* = 0.471, η^2^ = 0.031). Day-to-day comparisons showed that there was a day effect (*p* = 0.014, η^2^ = 0.235). CK increased on Tuesday morning (*p* = 0.009) following Monday’s HIFT session. CK returned to baseline on Wednesday morning before rising again on Thursday morning following Wednesday’s HIFT session (*p* = 0.022). CK on Friday morning was significantly lower than Thursday (*p* = 0.003) but not lower than Monday’s baseline (*p* = 0.594). Overall, CK rose after an HIFT session, and although statistically significant, this was within the normal healthy range.

With regard to C-reactive protein (CRP) concentration, this did not appear to be affected by training (Table 3). There was no group effect (*p* = 0.865, η^2^ = 0.002) nor week (*p* = 0.212, η^2^ = 0.090), day (*p* = 0.268, η^2^ = 0.073), or group × week × day interaction (*p* = 0.182, η^2^ = 0.101). CRP was also within the healthy range.

The compliance with the formula was confirmed by the fatty analyses.

## 4. Discussion

The aim of the present study was to investigate the potential effects of a combination of specific PUFAs and antioxidant vitamins on various exercise and performance parameters and inflammation markers over an 8-week HIFT period. The results showed that the multinutrient combination did not affect any aspect of performance following a period of HIFT. However, there was a significant effect on body composition, as indicated by the small but significant increase in estimated muscle mass and the decrease in fat mass only in the SG.

The beneficial effect of the multinutrient combination on body composition during HIFT is an important finding of the present study and this effect may be due to the ω-3 PUFAs present. However, the available information in the literature on this is equivocal, and there is no consensus on changes in muscle protein synthesis and lean body mass following the supplementation of omega fish oils. Under certain circumstances, like in elderly populations, omega fish oils appear to improve muscle protein synthesis [15,35] or to limit muscle loss [36]. In young healthy adults, ω-3 PUFA supplementation was effective in conditions of hyperinsulinemia and hyperaminoacidemia [37]. The combination of exercise and ω-3 PUFA supplementation has also proven beneficial for increasing muscle protein synthesis and type II fiber hypertrophy in elderly females [38]. Most of the positive effects of ω-3 PUFAs have been obtained in studies on elderly participants. In contrast, a recent study failed to show any additional effect of fish oil supplementation on muscle mass following 10 weeks of strength training in young adults [39]. In that and other studies, exercise itself appeared to have provided a sufficient stimulus for protein synthesis, and ω-3 PUFA supplementation did not elicit further increases in muscle protein synthesis and muscle mass [39,40]. Therefore, according to our knowledge, the present study is the first to show that a large dose of EPA and DHA can augment the hypertrophic stimulus during 8 weeks of HIFT in young healthy individuals. Still, one must note that the reasons for the discrepancy in the literature regarding the potential effects of ω-3 PUFAs on muscle protein synthesis and muscle mass are not clear yet, and further research is needed. The physiological mechanism behind our findings is beyond the scope of the present study, and no safe hypotheses can be made, considering the inconsistencies that are evident in the scientific literature.

Explosive strength improved similarly in both groups, with increases being slightly higher in SJs than CMJs (15.5% and 12.7% in SJs vs. 11.3% and 8.5% in CMJs). Part of this improvement in explosive strength may be due to neural adaptations, as well as due to muscle mass increases [39]. ω-3 fatty acids integrate into nervous tissue cell membranes [41], promoting the development of brain function and neural pathways [42], but ω-3 PUFAs can also enhance neuromuscular recruitment [43].

Findings on the effects of ω-3 PUFA supplementation on cardiorespiratory parameters are also inconclusive in the literature. The dosage and duration of the supplementation and the training level of the participants could all have played a part in that inconsistency [44]. Jost et al. [45] supplemented male amateur runners for 12 weeks with 2234 mg of EPA and 916 mg of DHA, and they did not find greater improvement in VO_2_peak with these men compared to the control group. Oostenbrug et al. [46] supplemented 24 well-trained male cyclists with 6 g·day^−1^ for 3 weeks, with or without vitamin E (300 IU·day^−1^), and did not find any effect on VO_2_max or time-to-exhaustion testing. On the other hand, in a study by Zebrowska et al. [13], a small dose of 1.3 g·day^−1^ ω-3 PUFAs for three weeks led to an increased VO_2_max in well-trained cyclists, which was attributed to an improved endothelial function. In the present study, despite a greater daily dosage of ω-3 PUFAs compared to other studies, both groups similarly improved VO_2_max. ω-3 fatty acids have been shown to have several positive physiological effects that may contribute to a lower oxygen demand, such as increased endothelial nitric oxide production [47], increased insulin sensitivity [48], a shift to glucose utilization by the muscle with less oxygen consumption compared to fat metabolism [49], and improved calcium handling in the skeletal muscle [50]. Still, a performance effect cannot be warranted based solely on biochemical changes seen within the muscle cells following ω-3 PUFA supplementation, and further research on this is required [14].

The supplement used in the present study was also rich in antioxidant vitamins A and E and γ-tocopherol. The chronic supplementation of antioxidants may reduce or blunt endurance training adaptations [51,52]; vitamin E supplementation at high doses should not be recommended for healthy individuals [53], and only individuals deficient in a certain antioxidant may benefit when supplemented according to their individual needs [54], although there was not any screening on plasma vitamin concentrations in the present study. It may be argued that the SG in the present study could have had a further increase in VO_2_max if they had not been supplemented with antioxidant vitamins. The increase in VO_2_max observed in the present study was comparable to that of other HIFT studies of similar modality and duration [3,55], while VO_2_max improved similarly in both the supplement and placebo groups. Therefore, we can safely say that the inclusion of vitamin A and vitamin E in the present study did not have any adverse effects on cardiovascular training adaptations and VO_2_max.

CK and CRP values were measured daily during the first and eighth weeks of the training period. The results showed that CK rose the morning after a training session and declined in the next 24 to 48 h, following an expected pattern [56]. CK rises following intense exercise due to increased membrane permeability or muscle damage [57], depending on exercise intensity [58]. There are data in the literature showing that supplementation with ω-3 PUFAs may reduce exercise-induced muscle damage, aid in recovery, and potentially promote the recovery of performance [44]. In the present study, there was no difference between the supplement and placebo groups. This discrepancy could be attributed to the fact that the studies showing a positive effect of ω-3 PUFAs on muscle damage used an exercise program designed specifically to elicit muscle damage such as downhill running [59] and eccentric strength exercise [60,61]. In the present study, we used a reasonable HIFT protocol in order to have performance improvements, not purposely targeting muscle damage. Furthermore, in the present study, CK values rose statistically, but the increase was only ~1.40-fold. It has been shown that CK values in physically active individuals are about twice as high compared with sedentary individuals [56,62]. One other aspect, often overlooked, is that in some experiments, ω-3 PUFA supplementation was initiated a month earlier than in the exercise trial, and this may explain the beneficial effects observed in these studies following damaging eccentric muscle contractions [61]. In contrast, when supplementation starts simultaneously with the exercise program, supplementation does not appear to ameliorate inflammation or muscle damage or accelerate recovery and exercise performance [63]. Inflammation in the present study was monitored via CRP analyses. Statistical analysis showed that there was no change from day to day between groups or between the beginning and end of the training program. Again, this may be attributed to the HIFT modality used in the present study, which did not elicit excessive inflammation and deems HIFT a safe and effective type of training to be used for fitness improvement.

### Limitations

In the present study, nutrition was not strictly controlled. The participants were asked to maintain their normal diets during the intervention period. There was frequent communication with the participants, reminding them to adhere to the researchers’ guidelines. Since there was no change in body mass, we can, therefore, assume they adhered to our guidelines.

Body composition was assessed with bioimpedance, using the latest available multifrequency technology. Still, there are some measurement errors with this method, compared to DEXA, which is the gold-standard method. Precautions were taken to minimize potential errors, the participants followed a similar food and fluid intake 24 h prior to both body composition assessments, and all guidelines for body composition assessments were followed.

The supplement given in the study contained antioxidant vitamins. However, we did not measure oxidative stress markers before and after the intervention as it was not the aim of this study.

## 5. Conclusions

HIFT combined with high doses of ω-3 PUFAs and antioxidant vitamins as a supplement results in a small but significant gain in muscle mass and fat reduction compared with HIFT alone. Supplementation with antioxidant vitamins neither impairs nor promotes the cardiorespiratory performance benefits of HIFT. HIFT with or without supplementation is effective in inducing beneficial changes in body composition, muscle strength, and cardiorespiratory fitness without causing inflammation or considerable muscle damage. Further studies are needed to measure muscle protein synthesis and to identify potential physiological mechanisms for the beneficial effects of combining HIFT with ω-3 PUFAs, ω-6 PUFAs, and antioxidant vitamins.

## Figures and Tables

**Table 1:** Anthropometric and body composition data of the experimental (SG) and the placebo groups (PG) before and after the intervention. *: significantly different from baseline.

Variable	SG (*n* = 10)		PG (*n* = 9)		Interaction and Main Effects (*p*-Value)		
	Before	After	Before	After	Time × Group	Time	Group
Body mass (kg)	65.7 ± 10.3	65.7 ± 9.6	64.2 ± 10.3	63.6 ± 10.2	0.393	0.277	0.700
Body mass index (kg m^−2^)	23.0 ± 2.5	22.8 ± 2.5	23.4 ± 1.9	23.1 ± 2.0	0.766	0.107	0.702
Βody fat (%)	21.0 ± 5.3	19.1 ± 5.3 *	24.0 ± 8.1	23.7 ± 7.4	0.030	0.004	0.220
Muscle mass (kg)	24.8 ± 5.8	25.2 ± 5.6 *	23.0 ± 6.3	22.7 ± 6.3	0.007	0.591	0.443

**Table 3:** Creatine kinase (CK) and C-reactive protein (CRP) morning daily values during week 1 and week 8 of the HIFT program.

CK (U·L^−1^)					
	Monday	Tuesday	Wednesday	Thursday	Friday
Week 1					
Supplement group	185 ± 136	257 ± 153 **	220 ± 138	290 ± 237 **	233 ± 163
Placebo group	184 ± 112	267 ± 171 **	203 ± 134	243 ± 155 **	188 ± 128
Week 8 *					
Supplement group	176 ± 160	201 ± 110 **	171 ± 96	199 ± 127 **	163 ± 82
Placebo group	106 ± 42	156 ± 72 **	112 ± 53	142 ± 66 **	113 ± 49
CRP (mg·dL^−1^)					
Week 1					
Supplement group	1.0 ± 0.7	0.9 ± 0.7	0.9 ± 0.8	2.7 ± 4.2	3.6 ± 1.1
Placebo group	1.8 ± 1.9	1.6 ± 1.6	1.4 ± 1.5	1.2 ± 1.5	1.6 ± 2.5
Week 8					
Supplement group	1.0 ± 1.4	1.0 ± 1.5	0.9 ± 1.3	1.1 ± 1.2	1.1 ± 1.3
Placebo group	1.1 ± 2.1	0.9 ± 1.5	0.9 ± 1.4	1.4 ± 2.3	1.1 ± 1.4

CK values in week 8 * were significantly lower than in week 1 (*p* = 0.009). ** denotes that CK increased compared to Monday’s baseline. Abbreviations: CK, creatine kinase; CRP, C-reactive protein.

## Data Availability

All data and materials of this study are available upon request due to ethical reasons.
